# Gastric Remnant Perforation 10 Years After Roux-en-Y Gastric Bypass: A Report of a Rare Case

**DOI:** 10.7759/cureus.85077

**Published:** 2025-05-30

**Authors:** Hugo Pereira, Daniel Martins, Mariana Santos, Amélia Tavares, Manuel Oliveira

**Affiliations:** 1 Surgery, Centro Hospitalar Vila Nova de Gaia e Espinho, Espinho, PRT; 2 General Surgery, Centro Hospitalar Vila Nova de Gaia e Espinho, Espinho, PRT

**Keywords:** bariatric surgery complications, gastric remnant perforation, helicobacter pylori, interdisciplinary collaboration, roux-en-y gastric bypass

## Abstract

Gastric remnant perforation is a rare yet potentially fatal complication following Roux-en-Y gastric bypass (RYGB). The exclusion of this segment from the alimentary tract creates significant diagnostic challenges, often leading to misdiagnosis. We report the case of a 42-year-old woman, 10 years status post RYGB for obesity, presenting with acute generalized abdominal pain. Initial imaging studies suggested ovarian torsion, but diagnostic laparoscopy revealed a perforation of the gastric remnant. Laparoscopic repair was successfully performed, and the patient recovered without complications. This case underscores the importance of maintaining a high index of suspicion for gastric remnant complications in post-RYGB patients, particularly when symptoms mimic alternative intra-abdominal pathologies. Early interdisciplinary collaboration and timely surgical intervention are crucial for optimal outcomes.

## Introduction

Obesity is a global public health challenge and a major risk factor for metabolic and cardiovascular diseases. It is characterized by excessive adipose tissue accumulation that impairs physical and metabolic health and is associated with type 2 diabetes mellitus, hypertension, cardiovascular diseases, obstructive sleep apnea, non-alcoholic fatty liver disease, and various malignancies such as colorectal, breast (postmenopausal), endometrial, and renal cancers [1,2].

Despite lifestyle modifications and pharmacological therapies, many patients with severe obesity fail to achieve sustained weight loss. In such cases, bariatric surgery remains the most effective long-term intervention [3,4].

According to international guidelines (e.g., National Institutes of Health (NIH), International Federation for the Surgery of Obesity and Metabolic Disorders (IFSO)), surgery is indicated in individuals with a BMI ≥35 kg/m² or ≥30 kg/m² with significant obesity-related comorbidities [5,6]. Roux-en-Y gastric bypass (RYGB) is widely regarded as the gold standard among bariatric procedures, owing to its dual restrictive and malabsorptive mechanisms that contribute to durable weight loss and improvement in obesity-related conditions [7,8,9]. However, this procedure is not without complications. Long-term adverse events include anastomotic ulcers (2-10%), strictures (4-10%), internal hernias, and, more rarely, complications involving the excluded stomach, such as gastric remnant perforation (<1%) [10].

The exclusion of the gastric remnant from the alimentary tract poses unique diagnostic challenges [11]. Its inaccessibility through routine endoscopy complicates the identification and timely management of complications. Although rare, perforation of the gastric remnant poses significant diagnostic and therapeutic challenges, with few cases reported in the literature, often diagnosed late due to nonspecific symptoms and complex post-surgical anatomy [12].

The aim of this case report is to raise awareness of gastric remnant perforation as a rare but potentially life-threatening late complication of RYGB and to emphasize the diagnostic difficulties resulting from altered postoperative anatomy.

## Case presentation

A 42-year-old woman presented to the emergency department with acute generalized abdominal pain of three days’ duration. The pain was progressively worsening, incapacitating, and associated with nausea and multiple episodes of vomiting. She had undergone RYGB a decade earlier, with significant and sustained weight loss and no prior postoperative complications. As a part of her preoperative workup, she underwent successful eradication of *Helicobacter pylori*. The patient denied smoking, alcohol consumption, and recent use of nonsteroidal anti-inflammatory drugs.

**Figure 1:**
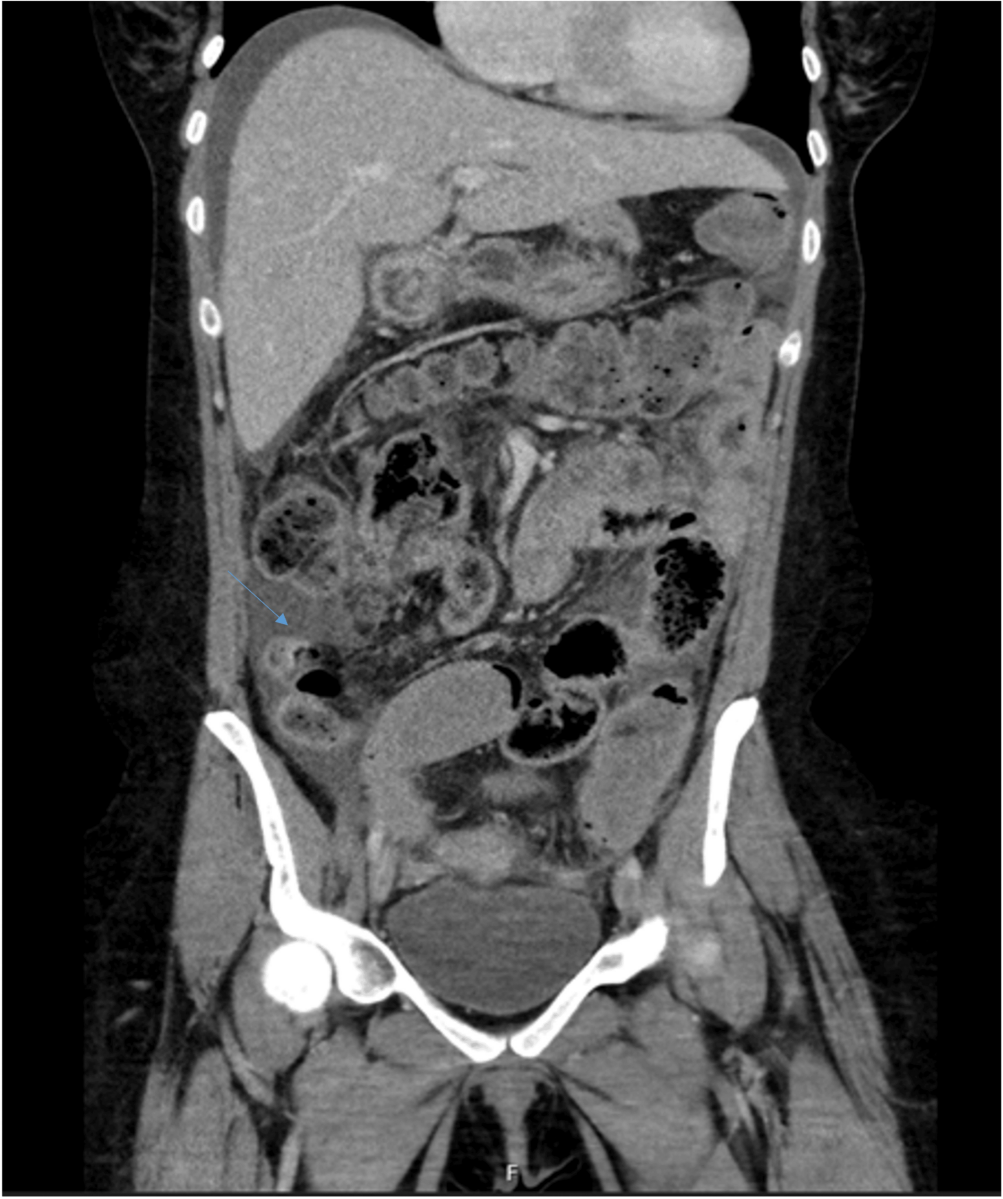
Coronal contrast-enhanced abdominal CT showing free intraperitoneal fluid (arrow), suggestive of a peritonitis (without pneumoperitoneum) in a patient with prior Roux-en-Y gastric bypass (RYGB).

On examination, she was afebrile and tachycardic but normotensive and did not require supplemental oxygen. There was generalized abdominal tenderness with signs of peritonitis. Laboratory tests showed no leukocytosis or elevated inflammatory markers. Computed tomography (CT) (Figure 1) of the abdomen and pelvis revealed free intraperitoneal fluid and a suspected left ovarian torsion. Diagnostic laparoscopy performed by the gynecology team identified a large ovarian cyst without torsion and significant free intraperitoneal fluid, prompting consultation with the general surgery team.

**Figure 2:**
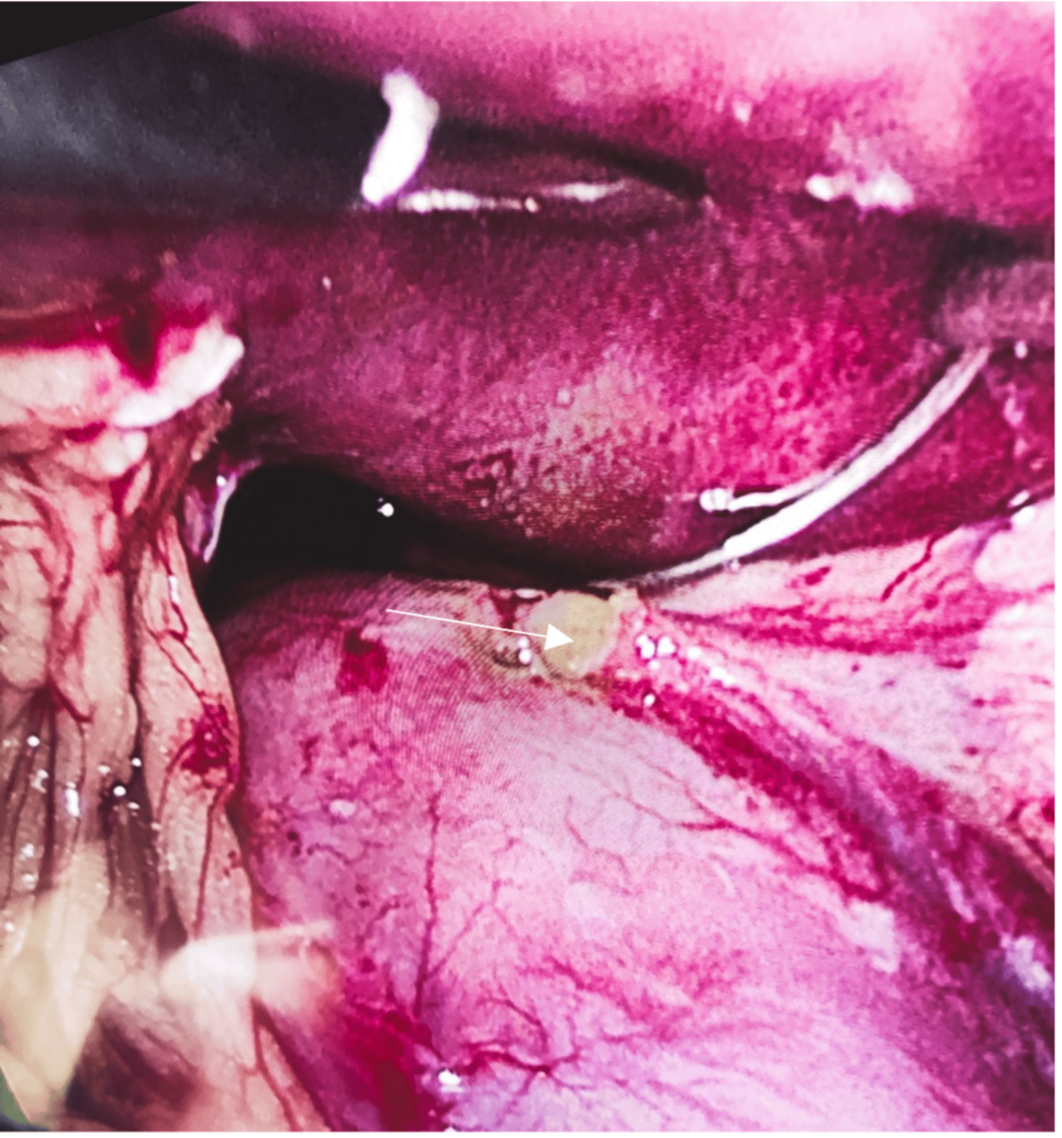
5 mm perforation on the anterior wall of the gastric remnant (arrow).

Subsequent laparoscopic exploration revealed a 5-mm perforation on the anterior wall of the gastric remnant (Figure 2). A biopsy was done, and the defect was repaired with an interrupted non-absorbable multifilament suture (silk) reinforced by an omental patch. The peritoneal cavity was irrigated thoroughly, and a drain was placed in the sutured area. The patient recovered uneventfully and was discharged on postoperative day 5 with instructions to avoid NSAIDs (nonsteroidal anti-inflammatory drugs). Postoperative testing of the biopsy for *H. pylori* was negative.

## Discussion

Gastric remnant perforation after RYGB is a rare but serious complication, with limited cases reported in the literature [11-12]. The excluded gastric remnant, isolated from the alimentary tract, presents unique diagnostic and therapeutic challenges. This case highlights the diagnostic complexity and the crucial role of interdisciplinary collaboration in both surgical decision-making and preoperative management.

Although uncommon, gastric remnant perforation should be considered in the differential diagnosis of patients with acute abdominal symptoms, particularly when imaging reveals free intraperitoneal fluid without pneumoperitoneum. The diagnosis of gastric remnant perforation is inherently challenging due to its inaccessibility via endoscopy and nonspecific clinical presentation. Acute abdominal symptoms are often the first indicators. Imaging studies, such as CT scans, are helpful but may fail to localize the perforation. In this case, the initial misdiagnosis as suspected ovarian torsion highlights how the atypical presentation of gastric remnant perforation can mimic other intra-abdominal pathologies, potentially delaying definitive treatment [13].

The involvement of multiple specialties was crucial in this case. The gynecology team's prompt decision to involve General Surgery specialists after detecting free fluid without an apparent gynecological origin exemplifies the value of interdisciplinary collaboration in managing intricate cases. This interdisciplinary approach facilitated timely diagnosis and surgical management, likely improving the patient’s outcome.

Successful bariatric surgery outcomes depend not only on technical precision but also on thorough preoperative preparation. One critical step in preparing patients for RYGB is the eradication of *H. pylori*, given the anatomical challenges posed by the surgery and the high prevalence of this bacterium in these patients. Since the remnant stomach becomes inaccessible via routine endoscopy, any future gastric pathology is difficult to diagnose and manage. Preoperative testing for *H. pylori *(via urea breath test, stool antigen test, or endoscopic biopsy) and eradication of positive cases are widely recommended in bariatric programs to minimize postoperative complications. *H. pylori* is a known risk factor in general, but was not present in this case, making the etiology more ambiguous [14].

In addition, the inability to monitor the remnant stomach reinforces the importance of preoperative* H. pylori *eradication in reducing the risk of gastric cancer. Studies indicate that the prevalence of *H. pylori *in bariatric candidates ranges from 20% to 60%, depending on the population [10], making preoperative treatment a key factor in reducing marginal ulcer formation and other complications.

In this case, histopathological examination revealed a perforated gastric wall associated with ulceration, areas of both acute and chronic inflammation, granulation tissue, and features consistent with ischemic and hemorrhagic injury, alongside reactive epithelial changes. No evidence of *H. pylori*, dysplasia, or malignancy was identified. This result underscores the challenges of managing remnant gastric pathology and highlights the need for a comprehensive approach to preoperative assessment and long-term follow-up. The etiology of gastric remnant perforation is multifactorial, often linked to marginal ulcers, ischemia, or staple-line dehiscence. Key risk factors include NSAID use, which compromises the gastric mucosal barrier and increases ulceration risk. However, this patient denied NSAID use, making it an unlikely cause. *H. pylori* infection, a well-established contributor to peptic ulcer disease, was also ruled out, as postoperative testing was negative. Chronic ischemia, resulting from altered gastric blood flow during surgery, remains a plausible explanation for the perforation in this case. In addition, although rare, staple-line erosion or dehiscence can occur years after surgery due to mechanical or metabolic stress [15].

Definitive treatment of gastric remnant perforation is surgical, typically involving laparoscopic or open repair of the defect. In this case, laparoscopic exploration allowed the identification and repair of the 5-mm perforation, minimizing surgical morbidity. The use of interrupted sutures reinforced with an omental patch is a well-established technique, providing structural support and promoting healing [16]. The thorough irrigation of the peritoneal cavity and placement of a drain are essential to manage contamination and prevent abscess formation. Postoperative care includes addressing underlying risk factors, such as avoiding NSAIDs, ensuring the absence of *H. pylori *infection, and eviction of alcohol and tobacco [13].

Long-term complications following RYGB extend beyond gastric remnant perforations and include internal hernias, nutritional deficiencies, and bowel obstructions. Internal hernias, often due to anatomical rearrangement during surgery, can cause intermittent or acute abdominal pain and require prompt surgical intervention. Studies estimate their incidence to range between 1% and 4% post-RYGB. [16]

Nutritional deficiencies are another major concern due to the malabsorptive nature of the procedure. Deficiencies in vitamin B12, iron, calcium, and fat-soluble vitamins can lead to anemia, osteoporosis, and neurological symptoms. Lifelong supplementation and regular monitoring are essential to mitigate these risks [17].

Bowel obstructions, although less frequent, remain a potential complication, typically caused by adhesions or strictures at surgical sites. These require careful diagnostic evaluation, often necessitating imaging and, in some cases, surgical intervention. A thorough understanding of these complications allows for improved postoperative care and patient outcomes [18].

Documented cases of gastric remnant perforation are sparse, with limited data available in the literature. Most reported cases occur within the first five years post-RYGB and are often associated with common risk factors such as NSAID use or *H. pylori* infection [13]. A systematic review by Gonzalez-Perez et al. found that the majority of gastric remnant perforation cases occurred in the early postoperative period and were linked to NSAID usage [16].

This case stands out due to its late presentation, occurring 10 years postoperatively, and the absence of these common risk factors. Dai L, Shah MM, and Rosenblatt S described a case of gastric remnant perforation that occurred several years post-RYGB, highlighting the importance of long-term monitoring and the atypical presentations that may arise in such patients [15].

While no comprehensive registry exists to quantify the exact number of cases, reviews and isolated case reports suggest that these events are exceedingly rare, with fewer than 100 cases reported worldwide. Each documented case contributes valuable insights into the mechanisms and management of this uncommon complication, emphasizing the need for ongoing research [15].

## Conclusions

Gastric remnant perforation, although rare, should be included in the differential diagnosis of RYGB patients presenting with acute abdominal pain, even many years after surgery. This case underscores the diagnostic challenges posed by altered anatomy, the importance of interdisciplinary collaboration, and the need for prompt surgical management. While classical risk factors such as NSAID use and *H. pylori *infection were absent in this patient, other mechanisms such as chronic ischemia or staple-line erosion should be considered. This highlights the need for ongoing research into less well-defined etiologies. Finally, continued reporting of such rare complications is essential to improving recognition, understanding of pathophysiology, and long-term outcomes in bariatric patients.
